# Comparative Assessment of the Reproductive Status of Female Atlantic Bluefin Tuna from the Gulf of Mexico and the Mediterranean Sea

**DOI:** 10.1371/journal.pone.0098233

**Published:** 2014-06-09

**Authors:** Jessica M. Knapp, Guillermo Aranda, Antonio Medina, Molly Lutcavage

**Affiliations:** 1 Department of Biological Sciences, University of New Hampshire, Durham, New Hampshire, United States of America; 2 Departamento de Biología, Facultad de Ciencias del Mar y Ambientales, Campus de Excelencia Internacional del Mar (CEI-MAR), Universidad de Cádiz, Puerto Real, Cádiz, Spain; 3 Large Pelagics Research Center, University of Massachusetts Amherst, Gloucester, Massachusetts, United States of America; Institut Maurice-Lamontagne, Canada

## Abstract

Despite attention focused on the population status and rebuilding trajectory of Atlantic bluefin tuna (*Thunnus thynnus*), the reproduction and spawning biology remains poorly understood, especially in the NW Atlantic. At present, the eastern and western spawning populations are believed to exhibit different reproductive characteristics and, consequently, stock productivity. However, our study suggests that the two spawning populations, the Gulf of Mexico and the Mediterranean Sea, could show similar reproductive features and spawning strategies. Between 2007 and 2009, gonad samples from female Atlantic bluefin tuna were collected in the northern Gulf of Mexico (n = 147) and in the western Mediterranean Sea (n = 40). The histological and stereological analysis confirmed that sampled eastern and western bluefin tuna exhibit the same spawning duration (three months) but the spawning in the Gulf of Mexico begins one month earlier than in the Mediterranean Sea. Western bluefin tuna caught in the peak of the spawning season (May) showed a similar spawning frequency (60%) to the spawning peak observed in the Mediterranean Sea (June). Fecundity for the Gulf of Mexico fish (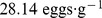) was lower but not significantly different than for fish sampled in the Mediterranean Sea (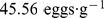). Our study represents the first comparative histological analysis of the eastern and western spawning stocks whose findings, combined with new determinations of size/age at maturity and possible alternative spawning areas, might suggest basic life history attributes warrant further scientific and management attention.

## Introduction

The reproductive biology of Atlantic bluefin tuna (ABFT; *Thunnus thynnus*, L. 1758) remains poorly understood despite the high economic value of this fishery and its exploitation throughout the Atlantic Ocean and the Mediterranean Sea. These uncertainties directly affect our understanding of the recruitment and productivity of the stock and could result in inefficient management of the fishery. The International Commission for the Conservation of Atlantic Tunas (ICCAT) manages the population as two stocks (eastern and western), separated at 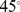
W based on two known spawning areas, the Gulf of Mexico (GMX) and the Mediterranean Sea (MED). This division assumes ABFT exhibit natal homing [1], [2] and have different maturation schedules [3], [4]. Nevertheless, electronic tagging and genetic/chemical marker studies have shown stock mixing on the foraging grounds indicating more complex population dynamics [1], [2], [5]–[7]. The eastern stock is estimated to be tenfold larger than the western stock [8]; consequently, the mixing rates are unbalanced with the eastern stock having greater influence on the western population, and any management action aimed at the eastern stock may indirectly affect the western stock [2]. Understanding the reproductive potential of both stocks is essential as it influences recruitment and hence the sustainability of the stocks and their capacity for supporting commercial fisheries [3],[9].

Electronic tagging and macroscopic examination of gonads are useful tools but, alone, are not sufficient for assessing population reproductive dynamics. Studies based on the histological analysis of gonads have allowed the characterization of the reproductive cycle of eastern spawners by the identification of maturation stages and estimations of several reproductive parameters [4], [10]–[13]. In the western and central Atlantic, these studies are scarce or impaired by large uncertainties [3], [9], [14]. Past studies examined gonad histology in fish from the Bahamas and the mid-Atlantic Bight and found a significant proportion of post-spawning females, but no ovaries with ovulated oocytes were observed [9], [15], [16]. However, these studies provided limited information since the samples were taken far from the known spawning areas and could not provide information about the reproductive condition of fish classified as active spawners [17], such as spawning frequency and/or fecundity [18].

While age at maturity in the eastern stock has been estimated as 3–4 y [4], [10], this parameter for the western spawning stock is the object of intense debate. ICCAT assumes an age at 50% maturity for the western stock of 8 y, but other studies provide estimations ranging from 4–16 y [9], [14], [19]–[21]. The younger maturation age observed in the eastern population [4], [9]–[11] leads to higher lifetime reproductive output and, consequently, larger productivity in the eastern than in the western stock.

Fecundity estimates allow the quantification of the reproductive capacity of individual fish and are essential for accurate assessment of the spawning stock biomass [22]. Assuming environmental characteristics between the MED and GMX spawning grounds are different, and ABFT exhibit natal homing, fecundity must be calculated separately for each stock.

An intensive evaluation of the maturity status of ABFT in the GMX compared with the MED would improve our comprehension of the reproductive connections between both stocks. In the present study, a histological and stereological analysis of gonads from ABFT caught in the GMX and the MED was undertaken to 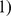 determine the reproductive status of female ABFT in the GMX through qualitative and quantitative histological studies, and 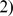 compare the reproductive parameters estimated between both spawning grounds.

## Materials and Methods

### Sample collection and biometry

Female ABFT were sampled from commercial fisheries in the GMX and the MED between 2007 and 2009. The National Marine Fisheries Service Pelagic Observer Program sampled fish from longline vessels in the northern region of the Gulf of Mexico from February–July (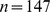). Eight samples obtained in February (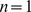) and March (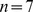) were not included in the stereological analysis because of low monthly sample size. In the MED, ABFT were sampled from longline fishing vessels on the western MED spawning grounds from mid June to mid July, 2008 (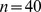). For all samples, curved fork length (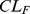) of each individual was measured to the nearest cm and converted to straight fork length (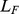) using the formula: 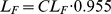 [23]. Body mass (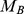) was calculated from 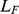 by location and timing of catch according to ICCAT conversions [23]. Ovaries were immediately removed and weighed, and the volume of the pair of ovaries (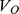) was estimated from their mass (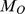) according to the equation 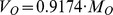 [12]. The gonadosomatic index (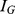) was calculated from 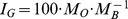.

### Histology

A subsample (0.5–1 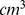) was removed from the central portion of one ovary and fixed for at least 24 h in 4% formaldehyde (10% formalin) in phosphate buffer, 0.1 M, pH 7.2. Tissue samples were dehydrated through increasing concentrations of ethyl alcohol, cleared, and embedded in paraffin wax. Samples were cut into 5–6 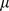
m sections using a microtome, and stained with either haematoxylin-eosin (GMX) or haematoxylin-VOF (MED) [23]. All images were taken on a Leica DMI 6000b using Image-Pro Plus.

**Figure 1:**
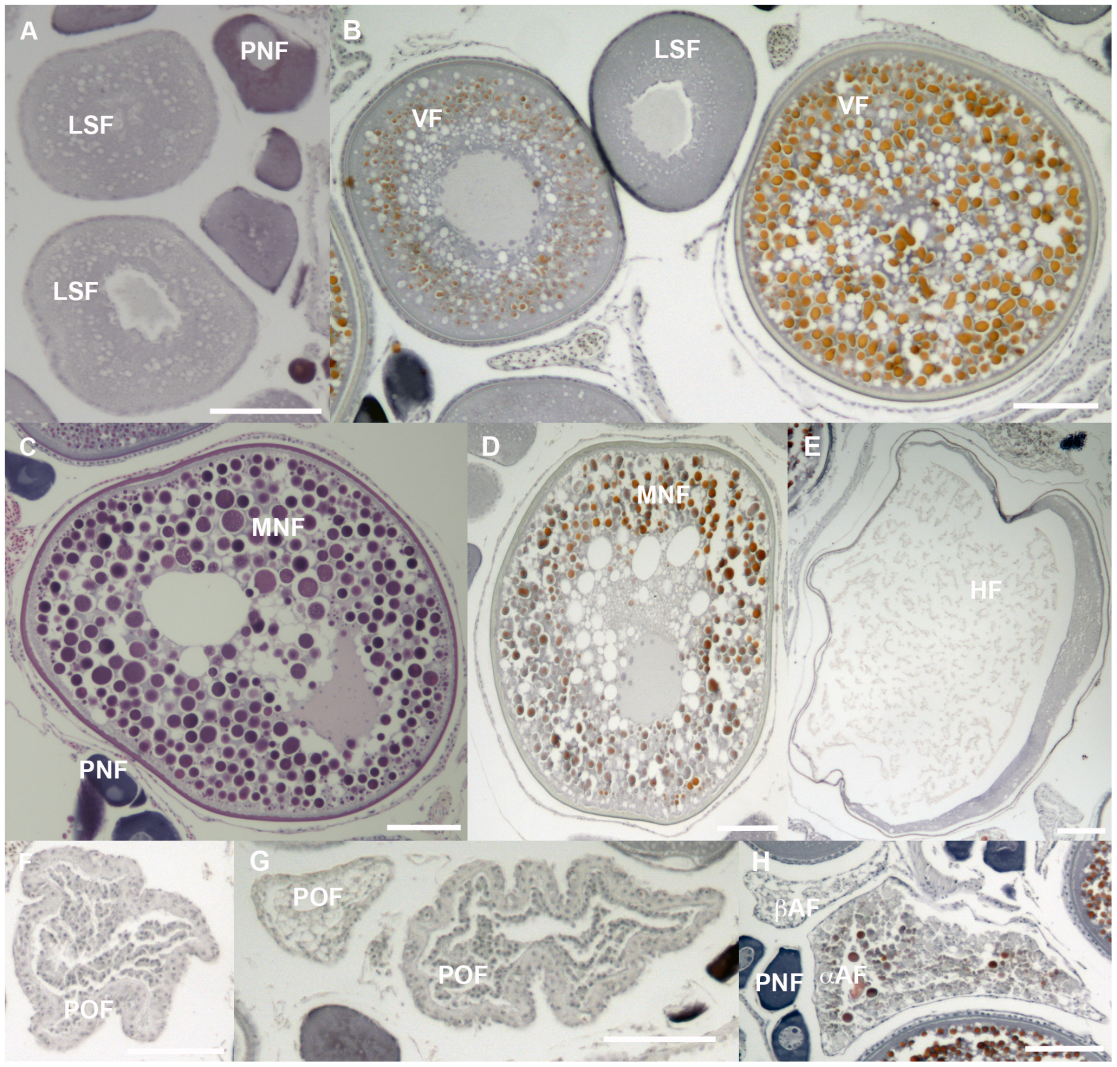
Stages of oocyte development observed in Atlantic bluefin tuna sampled from the Gulf of Mexico and Mediterranean Sea. A: lipid stage follicles (LSF); B: LSF, and early/late vitellogenic stage follicles (VF); C, D: migratory-nucleus follicles (MNF); E: hydrated follicle (HF); F, G: post-ovulatory follicles (POF); H: alpha (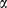
AF) and beta(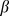
AF) atretic follicles. All scale bars are 100 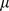
m.

Based on the oocyte development, eight distinct types of developing follicles were distinguished: perinucleolar (PNF), lipid-stage (LSF), vitellogenic (VF), and oocyte maturation follicles (OMF), which consisted of migratory-nucleus (MNF) and hydrated (HF) follicles. Additionally, 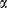
- and 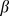-stage atretic follicles (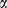
AF and 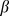
AF, respectively) and postovulatory follicles (POF) were counted (Figure 1). Based on the most advanced follicle type in the ovary and the extent of atresia, fish were classified as inactive (IN), active non-spawning (ANS), or active spawning (AS) [17], [25].

### Spawning frequency

Spawning frequency was estimated by the postovulatory follicle method [26] as adapted by [27]. This method calculates the mean spawning fraction as the total number of spawning females whose ovaries show postovulatory follicles (POF) divided by the total number of mature females sampled.

### Stereology

A stereological model-based method was applied to estimate numbers of the different categories of follicles according to the formula, 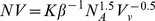 where 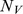 is the numerical density (number per unit volume) of the considered follicle type, 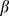 is a shape coefficient, *K* is a size distribution coefficient, 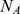 is the number of follicle transections per unit area, and 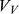 is the partial area (volume density). 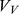 was calculated by analyzing 10 digital micrographs from each ovary using ImageJ [18], [28]. Values of both 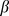 and *K* previously calculated for ABFT were used for this study [11], [12], [29]. The total number of follicles (*N*) was calculated by extrapolating 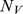 to the total ovarian volume, 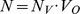. Ovarian volume loss through processing was measured, and corrections were applied, 34.8% for MED and 43.3% for GMX. Finally, the relative number of follicles (number of follicles per gram) was estimated as 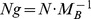.

### Statistical analysis

Comparisons of means of the stereological and biometrical parameters among years were performed using the Kruskal-Wallis test, and parameters with no significant difference were regrouped by month (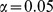). Monthly variation was also analyzed using the Kruskal-Wallis test (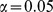). The Mann-Whitney U-test with Bonferroni correction was used to assess significant differences between pairs of months (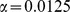) [30].

## Results

The 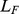 for fish sampled in the GMX (n = 147) was 172–326 cm, and 120–240 cm for fish sampled in the MED (n = 40). The 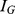 was 0.32–6.9 in the GMX and 0.3–5.8 in the MED. Mean 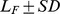 of fish sampled in the GMX (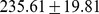 cm) was significantly larger (Mann-Whitney, 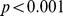) than those sampled in the MED (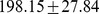 cm; Table 1). No significant differences in 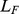 were observed within the GMX throughout the sampling period (Kruskal-Wallis, 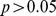). The mean 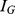 was higher in the MED than in the GMX (Mann-Whitney, 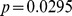), but this difference was only significant in June. Within the GMX, the 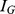 for females sampled in June was significantly lower than for those sampled in the other months (Kruskal-Wallis, 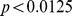; Table 2).

**Table 1:** Biometric data from Atlantic bluefin tuna, *Thunnus thynnus*, caught on longline fishing vessels operating in the Gulf of Mexico and Mediterranean Sea.

Spawning area	Year	Month	n	Straight fork length (cm)			*I_G_ (%)*
				Mean±SD	min.	max.	Mean±SD
GMX	2007	April–June	26	235.66±21.09	172	262	2.72±1.61
	2008	April–June	47	237.31±20.40	184	326	2.76±1.07
	2009	April–June	74	234.72±19.09	191	285	2.46±0.99
MED	2008	mid-June–mid-July	45	199.19±27.21	120	240	3.05±1.45

GMX = Gulf of Mexico; MED = Mediterranean Sea; 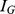 = gonadosomatic index.

**Table 2:** Stereological data for Atlantic bluefin tuna, *Thunnus thynnus*, sampled in the Gulf of Mexico and Mediterranean Sea.

		GMX									MED		
		n	April Mean±SD	n		May Mean±SD	n		June Mean±SD		n	June/July Mean_SD	
*(cm)*	SFL	32	241.35±25.27	a	100	233.08±17.80	a	15	241.30±16.28	a	42	198.12±27.61	b
	*I_G_*	27	2.76±1.20	a	68	2.67±1.09	a	12	1.75±0.76	b	39	3.05±1.45	a
*N_v_*	*α*ΑF	26	0.997±0.54		45	1.02±0.771		6	0.937±0.607		29	1.74±2.22	
	*β*AF	19	0.957±0.70	a	29	0.761±0.53	a	4	1.02±1.02	a,b	35	2.72±3.39	b
	LSF	29	23.40±7.28	a	82	19.25±7.97	a,b	13	18.22±10.03	a,b	41	17.58±10.82	b
	VF	29	5.05±1.74		83	6.00±1.79		13	5.76±2.37		36	6.29±2.18	
	MNF	0			9	0.47±0.29		2	0.41±0.50		4	0.48±0.34	
	HF	1	0.59		3	0.37±0.38		0			0		
	POF	9	1.40±1.09		45	1.22±0.93		5	1.06±0.54		24	1.46±0.94	
													
*N*	*α*ΑF	22	7.64±5.75		31	6.72±5.15		4	7.31±7.89		27	7.19±8.91	
	*β*ΑF	16	7.31±7.04		21	5.57±4.84		3	11.20±16.27	b,c	33	8.58±7.39	
	LSF	25	165.25±84.79	a	59	122.41±63.03	a,b	10	79.04±59.56		39	77.15±60.29	c
	VF	25	34.18±19.11		60	38.87±18.55		10	25.61±9.36		34	32.44±19.19	
	MNF	0			7	3.20±2.03		2	2.01±2.55		4	2.34±1.67	
	HF	1	4.89		2	0.829±0.108		0			0		
	POF	8	10.25±9.54		34	7.57±6.24		5	4.29±3.01		23	7.36±6.17	
													
*N_g_*	*α*ΑF	22	27.04±20.01		30	25.92±15.97		4	22.05±19.03		27	41.49±46.58	
	*β*ΑF	16	28.79±32.91	a	21	19.56±16.78	a	3	30.38±41.74	a,b	33	58.31±54.68	b
	LSF	25	579.15±248.21	a	58	455.30±216.89	a,b	10	282.62±214.98	b	39	462.75±318.73	b
	VF	25	120.90±61.39	a,b	59	144.59±59.77	a	10	91.92±26.75	b	34	200.64±99.33	c
	MNF	0			7	11.91±5.67		2	7.64±9.86		4	14.61±8.13	
	HF	1	23.63		2	3.00±0.53		0			0		
	POF	8	40.42±45.62		33	27.20±22.10		5	14.73±7.58		23	45.56±33.79	

Different letters in columns indicate significance.

GMX = Gulf of Mexico; MED = Mediterranean Sea; SFL = straight fork length; 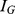 = gonadosomatic index; 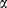
AF = alpha atretic; 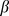
AF = beta atretic; LSF = lipid stage; VF = vitellogenic; MNF = migratory nucleus; HF = hydrated; POF = post-ovulatory follicles; 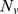 = numerical density (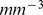); *N* = total number (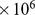); 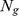 = relative number (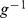).

### Histology

**Figure 2:**
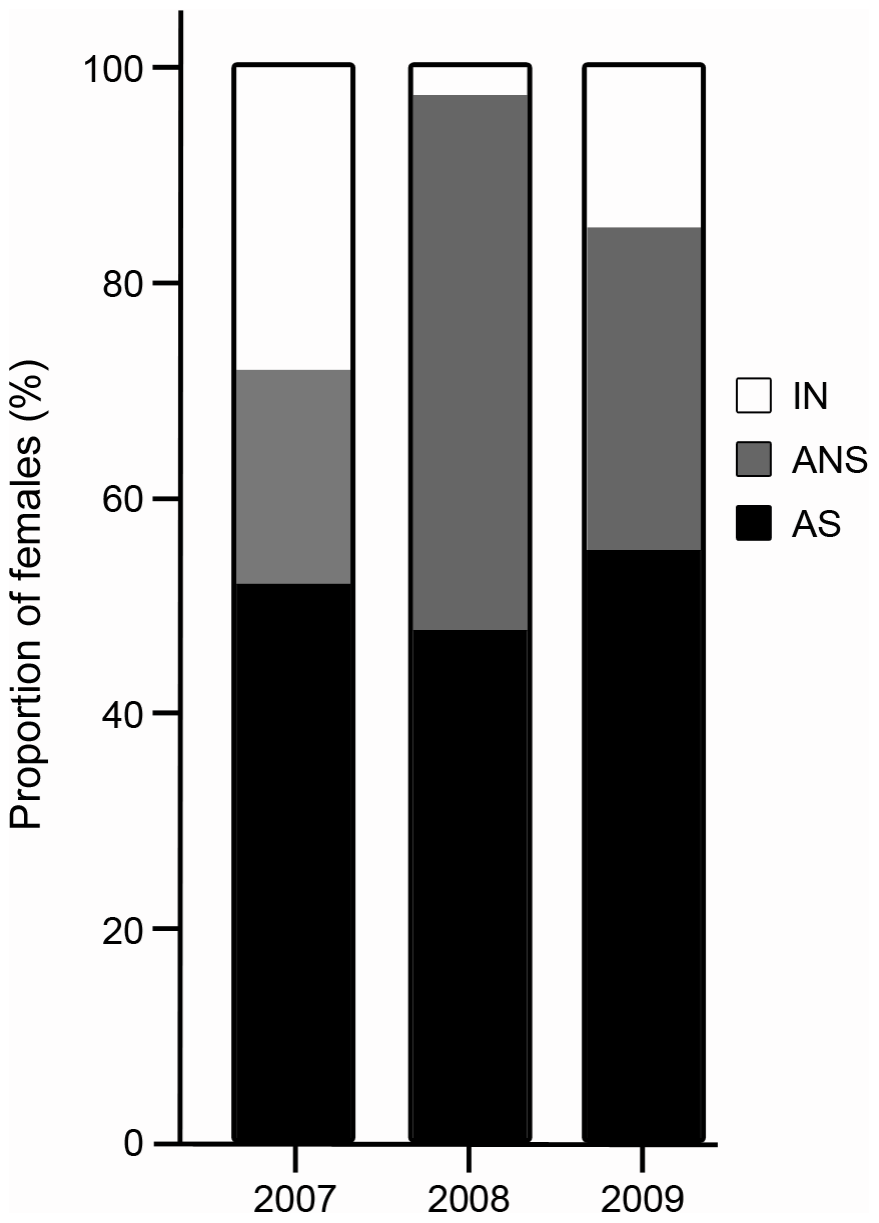
Maturity stages for female Atlantic bluefin tuna sampled in the Gulf of Mexico from 2007–2009. No significant difference was found between years for maturity stages, and thus, all years were pooled for future analyses. IN = inactive, ANS = active non-spawning, AS = active spawning.

Histological analysis of ovarian tissue from the GMX sampled from April to June showed no significant differences in the gonad development between years. The number of inactive (IN) females was less than 20% of our sample except in 2007 (28.0%). The proportion of active non-spawning (ANS) females was 20.0% (2007), 50.0% (2008) and 30.0% (2009). The proportion of active spawning (AS) females was consistent at about 50% for all years (Figure 2). Given the lack of annual variation, GMX samples were pooled for all years for subsequent analyses.

**Figure 3:**
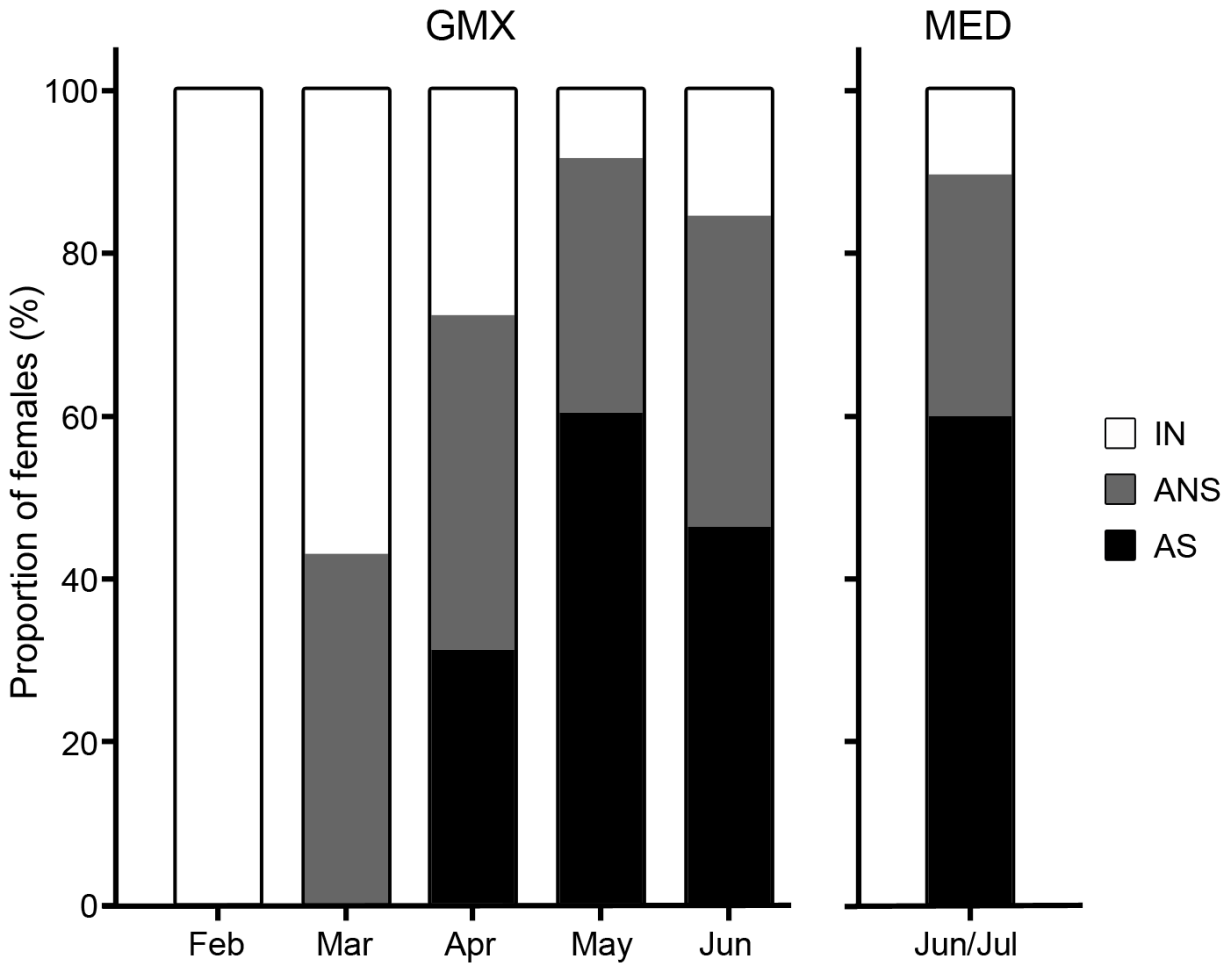
Maturity stages for female Atlantic bluefin tuna sampled in the Gulf of Mexico and Mediterranean Sea separated by month. No significant difference was found between years for maturity stages, and thus, all years were pooled for future analyses. IN = inactive, ANS = active non-spawning, AS = active spawning.

When ovary samples were arranged by month, consistent differences in the reproductive condition were observed with increasing maturation throughout the sampling period (Figure 3). In the GMX, the samples collected in February and March did not include any AS females. The proportion of AS females increased from April to June until a small decline was observed near the end of June. Near the end of the presumed spawning season in the MED (mid-June–mid-July), proportions of AS, ANS, and IN fish were similar to those observed in the GMX in May (Figure 3).

### Spawning frequency

Spawning fraction in the GMX was estimated to be 0.45 throughout the sampling period as 49 out of 108 mature females contained POFs in their ovaries. When this parameter was calculated by month, the proportion of females with POFs caught in April was lower than that in May and June. When the spawning frequency was calculated considering only AS females, its value increased significantly and remained similar among months. The spawning fraction in the MED samples was higher (0.60) than in the GMX (Table 3).

**Table 3:** Spawning frequency and spawning interval estimates for Atlantic bluefin tuna, *Thunnus thynnus*, sampled from the Gulf of Mexico and the Mediterranean Sea.

Spawning area	Month	Total	AS	With POFs	Spawning frequency		Spawning interval (days)
					(Total)	AS(only)	
GMX	April	30	12	9	0.30	0.75	3.33
	May	68	45	35	0.51	0.78	1.94
	June	10	7	5	0.50	0.71	2.00
	all months	108	64	49	0.45	0.77	2.20
MED	June/July	40	24	24	0.60	1.00	1.67

GMX = Gulf of Mexico; MED = Mediterranean Sea; AS = active spawning; POF = post-ovulatory follicle.

### Stereology

No significant differences were found between months for the stereological parameters of atretic follicles (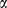
AF and 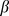
AF) in fish sampled in the GMX (Table 2). No significant differences were found for the numerical density of LSF (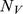
LSF) in the GMX despite the decrease observed throughout the reproductive season. Nevertheless, the total number of LSF (*N*LSF) and the relative number of LSF (*Ng*LSF) estimated in GMX fish in April was significantly higher than in June. In general, stereological counts of LSF quantified in the MED showed significantly lower values than those from the GMX sampled in April and May. Although the mean number of VF per 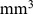 (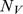
VF) and the mean number of VF (*N*VF) remained unchanged in the GMX, the relative number of VF (*Ng*VF) was significantly lower in June than in May. 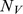
VF and *N*VF estimated in the MED were similar to the GMX values in June; however, because eastern fish were smaller than western fish on average, the relative number of VF (*Ng*VF) was much higher for MED fish. The low number of females with MNF and HF is likely the cause for finding no significant differences for these stages throughout the sampling period in the GMX. The highest number of POF (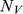
POF, *N*POF, and *Ng*POF) occurred at the beginning of the sampling period (April) and the lowest values were observed in June; however, no significant differences were found either among months or between the spawning areas. The realized fecundity estimated as the mean absolute number of POFs (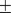
SD) was 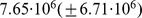 for fish from the GMX. This corresponds to relative batch fecundity (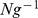) of 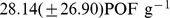. In the MED, the estimated mean number of POF was 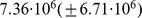 and the relative fecundity 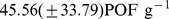 (Table 2).

## Discussion

Although not spatially and temporally exhaustive, this study represents the first attempt since the 1970s to accurately assess the spawning condition of ABFT sampled on the GMX spawning grounds and is the first histological and stereological comparison of the eastern and western spawning stocks.

Histological analysis of gonad samples from the spawning grounds throughout the spawning season is essential for evaluating the reproductive condition and performance of ABFT. Additionally, systematic sampling across the extent of the spawning grounds allows the study of temporal variations in key reproductive parameters, such as sex ratio, proportion of mature fish, spawning frequency, and spawning periodicity and fecundity. This information was lacking for the western stock resulting in large uncertainties for stock assessment and evaluation of productivity [8]. Since the implementation of the moratorium on directed fishing for ABFT in the GMX, federal fisheries observers have sampled ABFT caught as bycatch in the yellowfin tuna and swordfish longline fisheries (*Thunnus albacares* and *Xiphias gladius*, respectively). Such restrictions prevent comprehensive size sampling of spawning ABFT in the GMX and hinder the determination of the spatial and temporal extent of western spawning [31].

As a result of the bycatch sampling, previous studies have lacked small/medium fish (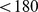 cm) leading to larger/older size and age at maturity estimates [20], [21]. Despite electronic tagging data that show presumed mature fish outside of known spawning areas [1], [32]–[34], these studies assumed western bluefin only spawn in the GMX. A New England study suggested previous ABFT sampling did not accurately represent the spawning size range of the western population because it only included fish sampled by longliners on known spawning grounds rather than all size classes sampled throughout their range [14]. Gear type, size selectivity, and vertical distribution of tuna by size also influence the size of spawners sampled by commercial fishing fleets [12], [35].

In this study, the smallest tuna sampled from the GMX, 172 cm and estimated age 7–8 y [36], had ripe ovaries with numerous recent POFs. This is consistent with an earlier study finding mature fish at 8 y [14] but not the recently proposed 12–16 y [20], [21]. In order to fully understand the reproductive dynamics of the western spawning stock, the maturity ogive should be revised using comprehensive size sampling over larger temporal scales including histological examination of the ovaries and endocrine profiling. New studies utilizing endocrine hormones developed and calibrated in captive eastern ABFT [37] indicate that western ABFT become sexually mature at less than 8 y [38].

Prior to the US moratorium, catch records indicated the presence of only giant ABFT (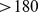 cm) in the GMX [3]. As opposed to the current management paradigm of western ABFT maturing at an older age than the eastern stock, fish may exhibit size and temporal segregation on the spawning grounds as is seen with the eastern spawning stock [3], [32], [39] and in Pacific bluefin tuna (*Thunnus orientalis*) [40]. There is indirect evidence that smaller fish may utilize alternative spawning locations, such as the Caribbean Sea, the Bahamas, or the Gulf Stream margins because ripe fish have been sampled there [9], [15], [41]. Given this evidence, it is possible that smaller bluefin spawn in alternative locations within the GMX as previous sampling has been concentrated in the north/central Gulf where US longline vessels operate. An ABFT life history model predicts that smaller/younger maturing fish should have shorter migration routes and spawn in areas closer to feeding areas than larger, older fish with higher energy reserves [42].

Electronic tagging results have consistently shown annual migration patterns of giant ABFT not entering either known spawning ground before returning to northern feeding grounds [1], [32]–[34]. It is possible that western ABFT spawn over a broader area of regions with oceanographic conditions appropriate for larval development than previously assumed [3], [14], [32], [34], [43]. Recent larval cruises found bluefin larvae outside the GMX [44], but spawning areas beyond the northern GMX await histological validation. Pop-up satellite tagged 2–5 year old ABFT did not enter the GMX or MED during presumed spawning periods (April–June) [45]. Nevertheless, some fish lingered in subtropical seas north of the Bahamas and in the southern mid-Atlantic Bight, areas visited by tagged adults [1], [5], [33], [46].

Our histological examination of the GMX bluefin ovaries revealed differences in follicle maturation between months throughout the spawning period. While samples collected in February and March contained no active spawning (AS) individuals, 31% of samples collected in April were AS individuals. As the spawning season progressed, the number of AS females increased and peaked in May (60%). The 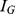 observed in June was significantly lower than other months indicating a decrease in ovarian size, and thus, the impending end of the spawning season for this region. The statistical results of the stereological analysis are consistent with previous findings and with the progression of the spawning season[11]. As the spawning season progresses, LSFs become less frequent indicating high levels of recruitment to VFs thereby compensating for losses caused by atresia or spawning. Similarly, the relative number of VFs (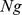
VF) was significantly higher in April and May than in June indicating a decrease in the recruitment of VFs as the end of the spawning season approaches.

In spawning fish, atresia is a natural mechanism for regulating the number of eggs spawned. Alternatively, massive atresia can indicate a cessation of oocyte maturation and/or spawning activity[47]. The GMX samples showed relatively low and stable levels of 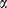
AF throughout the spawning season indicating ABFT found in the GMX are in favorable condition for oocyte maturation and spawning. While these fish appear to be actively spawning, tagged ABFT of presumed reproductively mature size observed outside the known spawning areas during the spawning period could be skipping spawning due to unfavorable body condition [14], [34]. ABFT sampled on the New England and Canadian foraging grounds have had periods of reduced somatic condition [48], [49] possibly accounting for an increased incidence of skipped spawning [50]. The incidence of skipped spawning in ABFT, however, is unknown, and modeling results show it is less likely to occur in larger, older fish in positive energy balance [42]. Giant ABFT sampled on western foraging grounds in the fall [14], [48] and in the MED in the spring and early summer [51] have extensive perigonadal fat and somatic lipid stores, and thus seem unlikely candidates for skipped spawning.

While ABFT have been observed on the western spawning grounds for months during the spawning season [34], individuals are believed to be actively spawning for only a few weeks [9], [52], [53]. These findings, albeit fishery-dependent, define the temporal borders of the reproductive events occurring in the north/central part of the GMX indicating the spawning season runs from April to June with maximum spawning activity in May. However, ABFT begin entering the GMX in late November, and those arriving in winter experience warm water masses of 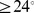
C in the lower GMX [34]. Reproductive sampling has been primarily conducted in the northern GMX and US territorial seas in late spring [9], [52]. Spawning activity occurring earlier in other areas of the GMX awaits confirmation by broader sampling, especially in Mexican territorial seas.

The relatively low proportion of GMX females with POFs in their ovaries (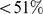) contrasts with the high spawning frequency (60%) observed in the western MED [12], [13]. The lower spawning frequency observed in the GMX could be the result of bias associated with utilizing the yellowfin and swordfish fisheries as the only sampling method. As long as bluefin reproductive studies rely on bycatch in commercial fisheries, it is not possible to obtain an unbiased, accurate assessment of ABFT reproduction. Given these constraints, it is important to note the temporal and spatial aspects of the sampling as well as the fishing gear used for any bluefin maturity study.

Stereological methods have often been used as an accurate tool for estimating fecundity in fishes, including eastern ABFT [11], [22], [29], [54], [55]. Realized fecundity can be estimated through stereological counts of POFs, whereas the number of MNF is an estimation of the potential fecundity [29], [56]. In this study, the mean relative batch fecundity was calculated directly from stereological counts of POFs and showed a decrease as the season progressed. This is atypical for indeterminate spawners [57], [58] but is likely due to the selective nature of sampling bluefin as bycatch in a longline fishery. One study suggested that monthly variation of fecundity may be masked by a decrease in the condition factor of fish appearing later on the spawning ground [59]. Although significant differences were not found between months, the highest value in the relative fecundity occurred early in the season (April), even though the number of AS females was still quite low. ABFT entering the GMX early might exhibit higher reproductive potential than those arriving later due to the good condition acquired on the foraging grounds. Otherwise, the lower spawning frequency observed on the western spawning grounds could be a consequence of migration distance [42] or decreased body condition observed on the western foraging grounds [48]. While being the first to arrive on the spawning grounds might provide increased resource availability for offspring, arriving in poor condition could decrease larval survival rates [51], [60].

The fecundity of eastern spawning bluefin was estimated at 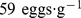 [12] and 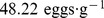 [13] for potential and realized fecundity, respectively. Our results show a lower realized fecundity for western spawners (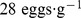) than eastern spawners; however, this difference was not statistically significant. This is in agreement with previous work indicating realized fecundity is not proportional to body size [13]. Although the differences in fecundity and maturity schedules of both ABFT stocks can be masked by sampling bias and complex population dynamics, fecundity has been shown to vary within a given species as a result of different adaptations to environmental habitats [61]. The unexpected variation of fecundity shown among large ABFT could be related to the environmental adaptations and the balance between body condition and energetic expenses during migration [42], [51].

Eastern and western ABFT spawning sites seem to exhibit the same periodicity (three months), but spawning in the northern GMX occurs one month earlier than in the western Mediterranean spawning ground. This is likely due to specific oceanographic conditions and the early warmer temperatures observed in the GMX (data from CCA-UNAM). In this study, we have observed similar values in bluefin reproductive parameters showing that the spawning condition of Mediterranean spawners from mid-June to mid-July is comparable with the reproductive peak observed in the GMX in May. Depth and temperature associations of electronically tagged ABFT entering the GMX in winter also suggest that it also serves as a foraging ground [34], as it does for other tunas and billfish species.

A more holistic view of the population dynamics of ABFT requires life history characteristics, reproductive profiles, and spawning areas and periodicity to be well defined, especially since they undoubtedly will change with shifts in climate and ocean productivity [62]. The extent and quality of lipids acquired by tunas before they arrive in spawning areas will affect eggs and larvae, and therefore, overall stock biomass [42], [51]. Future work should address energetic relationships between reproduction, migration, and early life history through modelling, biological sampling, and the development of smart tags to detect actual spawning events.
